# Improved Dynamic Mode Decomposition and Its Application to Fault Diagnosis of Rolling Bearing

**DOI:** 10.3390/s18061972

**Published:** 2018-06-19

**Authors:** Zhang Dang, Yong Lv, Yourong Li, Guoqian Wei

**Affiliations:** 1 Key Laboratory of Metallurgical Equipment and Control Technology, Ministry of Education, Wuhan University of Science and Technology, Wuhan 430081, China; dangzhang@wust.edu.cn (Z.D.); liyourong@wust.edu.cn (Y.L.); Weiguoqian@wust.edu.cn (G.W.); 2 Hubei Key Laboratory of Mechanical Transmission and Manufacturing Engineering, Wuhan University of Science and Technology, Wuhan 430081, China; 3 National Demonstration Center for Experimental Mechanical Education, Wuhan University of Science and Technology, Wuhan 430081, China

**Keywords:** dynamic mode decomposition, optimal rank truncation threshold, dominant modes selection strategy, fault diagnosis

## Abstract

To solve the intractable problems of optimal rank truncation threshold and dominant modes selection strategy of the standard dynamic mode decomposition (DMD), an improved DMD algorithm is introduced in this paper. Distinct from the conventional methods, a convex optimization framework is introduced by applying a parameterized non-convex penalty function to obtain the optimal rank truncation number. This method is inspirited by the performance that it is more perfectible than other rank truncation methods in inhibiting noise disturbance. A hierarchical and multiresolution application similar to the process of wavelet packet decomposition in modes selection is presented so as to improve the algorithm’s performance. With the modes selection strategy, the frequency spectrum of the reconstruction signal is more readable and interference-free. The improved DMD algorithm successfully extracts the fault characteristics of rolling bearing fault signals when it is utilized for mechanical signal feature extraction. Results demonstrated that the proposed method has good application prospects in denoising and fault feature extraction for mechanical signals.

## 1. Introduction

Rolling bearing plays an important role in modern equipment [1], relating to the health condition and the remaining service life. Amplitude-modulated and/or frequency-modulated (AM-FM) multi-component signals would be introduced when local damage such as cracks and exfoliation corrosion occurred during the operation [2]. It becomes intractable to extract the fault feature as the fact of that the measured signals are usually immersed with intensive background noise, especially in the incipient failure. Thus, robust fault feature extraction method should perfectly eliminate the noisy components and acquire the useful features generated from the non-stationary time series, which provides the proof for the maintenance plan before the whole machine gets disabled.

Recent decades have witnessed significant advances in the feature extraction of fault data collected from either experiments or numerical simulations. Short-time Fourier Transform (STFT) [3] adopts the method of piecewise averaging of the unstable signal. Several segments are sliced by a constant sliding window in accordance with the order of the original timeline. Spectrum characteristics are then received by Fourier Transform (FT) on each segment. However, the time-frequency resolution is constant and cannot transform over the change of time and frequency, which is the shortcoming of STFT. Wavelet Transform (WT) [4], a method of local time and frequency conversion, was proposed to represent the original signal with a mother wavelet by scaling and translation operation. While it can effectively extract information with multiscale refinement analysis, WT has an inherent problem of low frequency resolution of high frequency band. Wavelet Packet Transform (WPT) [5] was an extension of WT. Its basic idea was to make information energy concentration and decompose the signal into low-frequency components and high-frequency components. With further decomposition of the high frequency part, the time-frequency resolution of WPT is higher than that of WT. However, the choice of mother wavelets for WPT is hard to determine. Empirical Mode Decomposition (EMD) [6] was proposed to decompose the original time series to different intrinsic mode functions (IMFs) by means of adaptive low frequency and high frequency components. EMD is a method of adaptive signal processing that can process nonlinear and non-stationary signals. However, the EMD algorithm has many imperfect problems that need to be solved and improved, such as the effect of the endpoint and mode aliasing problems [7]. At the same time, the algorithm lacks physical significance. Singular value decomposition (SVD) [8] can also be used in processing the noise-corrupted signal, but the decomposition effect differs from the various choice of singular values. SVD may not be helpful when the noise energy reaches a certain level [9].

Dynamic mode decomposition (DMD), stemming from the fluid mechanics community [10], has been characterized as a powerful tool for Koopman spectrum analysis technology based on equation-free and data driven methods. It can extract the spatiotemporal coherent characteristics by decomposing the complex flow field into a series of simple expressions based on its inherent space-time. Recently, DMD is successfully used for prediction, state estimation and complex system control in the field of fluid mechanics [11]. Schmid [12] firstly defined the DMD algorithm by demonstrating that the theory was capable of describing the internal mechanism based on the numerical simulation and measured experimental data in a high-dimensional flow field. The core of the DMD algorithm can be regarded as a space dimension reduction technique, similar to the algorithm of proper orthogonal decomposition (POD). While POD decomposes data into a series of multiple orthogonal modal frequency components, and ranks them in terms of energy content, the DMD algorithm can describe the dynamic characteristics in a series of single non-orthogonal frequency modes and superpose them with coefficients according to the time scale [13]. Rowley [14] pointed out that DMD was closely related to the Koopman operator theory, a linear but infinite-dimensional operator that represented the action of a nonlinear dynamic system. Thus, DMD is applicable to deal with nonlinear systems such as roller bearing signal (which we attempt in this paper) based on the consideration that DMD is a numerical approximation to Koopman spectral analysis. The standard DMD algorithm for mechanical time-series signal processing is outlined in Section 2 with the theory provided by literature [12]. Since the appearance of the DMD algorithm, widespread use and great achievements have been made in the field of fluid application [11,13,14,15,16,17,18,19,20]. Nowadays, research regarding DMD mainly focuses on two aspects. Firstly, noise suppression algorithms were presented to correct the decomposition results due to the influence of noise. DMD cannot accurately extract dynamic characteristic information when the snapshots, periods of small data, are immersed by noise. These strategies range from a noise-corrected dynamic mode decomposition with control (ncDMD) [15], forward-backward DMD (fbDMD) [16], total least squares DMD (tlsDMD) [17], compressed DMD (cDMD) [18], and sparsity-promoting DMD (spDMD) [19]. Though most of the varietal DMD algorithms can extract fluid dynamic information more accurately, they are not self-adaptive as they need to give a rank truncation order in their processes. Secondly, it aims at computational complexity reduction in pre-processing and/or post-processing when big data are processed. This problem does not apply to the mechanical signals, since the amount of mechanical signals is usually smaller relative to the fluid domain.

Whether the DMD reconstruction matrix best approximates the original system dynamics’ characteristics largely depends on two aspects: the optimal rank truncation order of the companion/similarity matrix and the dominant modes selection strategy. In a standard DMD algorithm, a certain rank order r of the similarity matrix needs to be determined for POD decomposition. It will increase the calculation time if over-estimating the r value because of a large amount of data. Simulation results showed that the cross-correlation coefficient between the reconstruction data and the original synthetic signal even get a bit smaller along with the increase of the selected r. Conversely, the calculation result may not be accurate if the r value is too small, which would deduct some useful signal characteristics. In the past, most scholars presented the rank truncation criteria based on SVD, including selecting an “elbow” of the singular value with a logarithmic curve and a threshold such as a certain proportion of selected data variance. They mainly use the idea of discarding noises by substituting a smaller (compressed) matrix that captures the essential characteristics of the signal. A recent breakthrough by Gavish [21] provided a hard optimal threshold foundation in theory whether the noise magnitude is known or unknown. The methods of dominant mode selection strategy with a reduced order model are not identical. For a review of criterion to select dominant modes, we refer the reader to [22], which summarized the recent research situation and analyzed their respective advantages and disadvantages. At the same time, Kou [22] developed a method that multiplied each normalized DMD mode by its time coefficient. This method considered the evolution of each mode, but neglected the faint modes that should be treated as noise modes. Dang [23] introduced a tool, multiscale permutation entropy, to calculate the complexity of each DMD mode. Modes whose entropies were smaller than a threshold were chosen to recover the signal. However, for filtering the noise components, an entropy threshold needs to be set by multiple attempts.

In this paper, a DMD framework for mechanical fault signal processing is proposed, solving the intractable problems of optimal rank truncation threshold and dominant modes selection strategy based on the conventional DMD algorithm. The optimal rank truncation r of the similarity matrix is calculated with parametric non-convex penalty function for low-rank approximation. Inspired by WPT, which splits the signal up into a collection of different signals according to its level of decomposition, DMD modes are divided into slow modes and fast modes by splitting the continuous-time eigenvalues of the similarity matrix. Hierarchical application of the basic DMD algorithm is applied with a given level l. DMD modes are reconstructed by a number of bottomed modes. Fast Fourier Transform (FFT) is performed on the reconstructed signal for the purpose of extracting the fault features. The improved DMD algorithm is applied to process the rolling bearing’s simulation signal for noise reduction and feature extraction. Amazingly, by comparing the signal-to-noise ratio (SNR), our method has tremendous advantages in noise removal and fault feature extraction, in comparison with traditional mature noise reduction methods such as EMD, SVD and WT. Then, the proposed method is applied to the bearing signal in practice; as a result, it can successfully extract the fault characteristics that are highly consistent with the actual field.

This paper is organized as follows. Section 2 introduces the basic DMD algorithm for mechanical fault signal processing. Section 3 measures the solving of the optimal rank truncation order of the similarity matrix, and the strategy of dominant modes selection are outlined. Analysis results of dynamic simulation signal and practical bearing fault signal are described in Section 4. Conclusions are summarized in Section 5.

## 2. DMD Algorithm for Mechanical Signal Processing

DMD is an equation-free Koopman frequency analysis technique based on the theory of SVD and mode decomposition. Essentially, it is a kind of order reduction method that can represent potential dynamics’ characteristics of a complex high-dimensional system by extracting a series of single frequency. An order reduction algorithm decomposed from DMD in flow fields is divided into two kinds of mathematical expressions. One introduces a companion matrix approximating to the infinite dimensional linear Koopman operator, and another presents a similarity matrix for a linear mapping operation on the singular values of POD. For detailed description of the two methods, we suggest reading [22]. Both methods are capable of implementing the core algorithm, but the latter is more commonly used in numerical calculations for its robustness. We generalize the DMD algorithm framework for mechanical time-series signal processing based on the similarity matrix.

Supposing that a size of N sample points is acquired by a sensor with equal intervals between two successive sampling points, we define the one-dimensional time series as $S=[x_{1},x_{2},\cdots x_{i}\cdots x_{N}],\;x_{i}\in R$, while $\Delta t=x_{i+1}-x_{i}$. S can also be made from a digital simulation signal. A m×n shift-stack Hankel matrix $X_{S}$ can be constructed by Equation (1):

(1) $$X_{S}=\left[\begin{array}{llll} x_{1} & x_{2} & \cdots & x_{n}\\ x_{2} & x_{3} & \cdots & x_{n+1}\\ \cdots & & & \\ x_{i} & x_{i+1} & \cdots & x_{n+i-1}\\ \cdots & & & \\ x_{m} & x_{m+1} & \cdots & x_{m+n-1} \end{array}\right]=\left[\begin{array}{cccc} | & | & & |\\ X_{1} & X_{2} & \cdots & X_{n}\\ | & | & & | \end{array}\right],X_{S}\in R^{m\times n}.$$

In order to achieve the maximum spatial and temporal complexity of the original noisy signal (As in Equation (7), When the matrix is close to the square matrix, the maximum characteristic frequency components can be obtained with SVD), the product of m and n should be as large as possible. According to the principle of inequality, the product achieves maximum when m and n are equal or close to each other. The dimension m is defined as follows:

(2) $$m=\left\{ \begin{array}{l} N/2\;\;\;\;\;\;\;\;\;\;\;\;\;if\;N=2z;\\ \left(N+1\right)/2\;\;\;\;\;if\;N=2z-1; \end{array}\right.$$

where z∈R is positive integer sequence that z=1,2,3,….

It is possible to arrange the n column vectors into two m×(n−1) data matrices:

(3) $$X=\left[\begin{array}{cccc} | & | & & |\\ X_{1} & X_{2} & \cdots & X_{n-1}\\ | & | & & | \end{array}\right],Y=[\begin{array}{cccc} | & | & & |\\ X_{2} & X_{3} & \cdots & X_{n}\\ | & | & & | \end{array}],X,Y\in R^{m\times (n-1)}.$$

DMD is algorithmically a regression by assuming an optimal local linear approximation for mapping the current data to the subsequent data. The best-fit linear operator A is then used in terms of these signal matrices as:

(4) Y=AX.

Apparently, the evolution of the sequence X and Y is determined by the eigenvalues of A for the linear system. It can also be taken as an operator to approximate the dynamic characteristics when the data are produced in nonlinear systems, such as bearing vibration signal.

This solution minimizes the error:

(5) $$\underset{A}{\arg\min}{\left\|Y-AX\right\|}_{F},$$

where ${\left\|•\right\|}_{F}$ is the Frobenius norm, given by

(6) $${\left\|C\right\|}_{F}={\left(\sum_{j=1}^{n}\sum_{i=1}^{m}C_{ij}^{2}\right)}^{0.5}.$$

In what follows, we outline the key steps of the DMD algorithm for mechanical signal processing.

1. Firstly, seek an invertible matrix decomposition by SVD on matrix X:

(7) $$X\approx U\Sigma V^{*},$$

where U and V are orthonormal, called the left and right singular vector, respectively, $U^{*}U=I,V^{*}V=I$. The symbol ∗ denotes the complex conjugate transpose. $\sum \in R^{p\times p}$ contains a number of non-zero singular values $\left\{\sigma_{1},\cdots ,\sigma_{p}\right\}$ by descending sequence in its diagonal.

The matrix A of Formula (4) may be obtained by the pseudoinverse of X:

(8) $$A=YV\Sigma^{-1}U^{*}.$$

2. It is quite normal that the matrix A contains such a large amount of data that leads to the calculating processing reluctant. Thus, we choose a given number of truncated rank r, and project it onto POD modes with the order of characteristic vectors. Thus, we have a similarity matrix $\tilde{A}$ as follows:

(9) $$\tilde{A}=U_{r}^{*}AU_{r}=U_{r}^{*}YV_{r}\Sigma_{r}^{-1},$$

where $\tilde{A}\in R^{r\times r}$. The eigenvalues and eigenvectors of A are then represented by those of $\tilde{A}$ as they process the same dynamical features.

3. Perform eigenvalue decomposition on the similarity matrix $\tilde{A}$:

(10) $$\tilde{A}=W\Lambda W^{-1},$$

where $W=[\omega_{1},\omega_{2},\cdots ,\omega_{r}]\in R^{r\times r}$ is the eigenvectors of similarity matrix $\tilde{A}$, and $\Lambda =diag([\lambda_{1},\lambda_{2},\cdots ,\lambda_{r}])\in R^{r\times r}$ is a diagonal matrix containing the corresponding complex eigenvalue $\lambda_{i}$.

4. Compute the reconstruction matrix of DMD. The evolution of the signal characteristics can be characterized by the similarity matrix. In addition, the *i*-th eigenvector of original operator is presented by the relevant feature vector of the similarity matrix:

(11) $$\phi_{i}=YV_{i}\sum_{i}^{-1}W_{i}.$$

Formula (11), the project DMD approximate solution, is often called standard DMD mode. By firstly rewriting for convenience $\varpi_{i}=\ln(\lambda_{i})/\Delta t$, the approximate solution of reconstruction matrix of DMD is then given by:

(12) $$X_{DMD}=\sum_{i=1}^{r}\phi_{i}\exp(\varpi_{i}\Delta t)b_{i}=\Phi \exp(\Omega t)b,\;X_{DMD}\in R^{m\times n},$$

where Φ is a matrix consisting of DMD modes $\phi_{i}$, $\Omega =diag(\lambda_{i})$ is a diagonal matrix whose enters are continuous-time eigenvalues of the similarity matrix $\tilde{A}$, b is a vector containing the initial amplitude of each mode, and $b=\Phi^{\Gamma }X$, Γ denotes the Moore–Penrose pseudoinverse.

5. Finally, signal reconstruction and feature extraction can proceed based on the reconstruction matrix $X_{DMD}$. We take the first column of $X_{DMD}$ as the recuperative signal that the length is just about half of the original signal. Then, the Fourier transform is applied on the recuperative signal for a spectrogram, determining whether there is a failure frequency.

## 3. Improved DMD Framework

### 3.1. Rank Estimation Based on Convex Optimization

As in Section 2, non-zero singular values $\left\{\sigma_{1},\cdots ,\sigma_{p}\right\}$ are arranged in a descending sequence when performing SVD on X. Singular spectrum analysis treats the ahead k values representing the useful signals, and the residual p−k orders for part of the noise. The difficulty lies in the determination of optimized rank k. It cannot perfectly restore the dynamic information with the method of singular spectrum or hard optimal threshold foundation for their truncated rank orders k are smaller than the ideal ones, which is proved in Section 4.1. Here, a convex optimization framework is introduced to estimate the best rank r for Equation (9).

The noise polluted Hankel matrix X can be supposed to be a combination of an expectation matrix and a residual noise matrix:

(13) $$X=X_{E}+X_{N},\;X,X_{E},X_{N}\in R^{m\times n},$$

where $X_{E}$ is the desired low rank matrix, and $X_{N}$ is a zero mean Gaussian white noise matrix. Note that for unitary matrices U and V, $\Phi (X)=\Phi (U^{T}XV)$. Define the sparseness-inducing regularization ${\left\|X-X_{E}\right\|}_{F}$ by adding a penalty term λΦ(X). The typical resulting low-rank approximation problem can be written as follows:

(14) $$\begin{array}{ll} \overset{\wedge }{X} & =\arg\underset{X_{E}}{\min}\left\{\frac{1}{2}{\left\|X-X_{E}\right\|}_{F}^{2}+\lambda \Phi (X_{E})\right\}\\ & =\arg\underset{X_{E}}{\min}\left\{\frac{1}{2}{\left\|\Sigma -U^{T}X_{E}V\right\|}_{F}^{2}+\lambda \Phi (U^{T}X_{E}V)\right\}\\ & =U\cdot \arg\underset{X_{E}}{\min}\left\{\frac{1}{2}{\left\|X-X_{E}\right\|}_{F}^{2}+\lambda \Phi (X_{E})\right\}V^{T} \end{array},$$

where λ is a positive regularization parameter balancing the least-square fitting term, $\Phi (X_{E})=\sum_{i=1}^{k}\phi (\sigma_{i}(X_{E});\alpha )$, ϕ is a sparseness-inducing function, $\sigma_{i}(X_{E})$ is the eigenvalue of $X_{E}$, and 0≤α≤1/λ. When the function ϕ(x) is represented by |x|, the convex issue of low-rank approximation problem for rank minimization turns to a typical nuclear norm problem [24].

Define that $\Theta (\Sigma ,\lambda ,\alpha )=\arg\underset{X_{E}}{\min}\left\{\frac{1}{2}{\left\|X-X_{E}\right\|}_{F}^{2}+\lambda \Phi (X_{E})\right\}$.

Equation (14) can be equivalently written as:

(15) $$\overset{\wedge }{X}=U\cdot \Theta (\Sigma ,\lambda ,\alpha )\cdot V^{T}.$$

Dang [24] and Selesnick [25] proved that parametric non-convex penalty function method can be more accurate than using nuclear norm in estimating non-zero singular values, when the signal contains a large noise level. Therefore, the non-convex penalty function is used to obtain the optimized estimation of singular value as the following equation:

(16) Θ(Σ,λ,α)=min{|Σ|,max{(|Σ|−λ)/(1−αλ),0}}sign(Σ).

In this paper, the optimal rank r of singular value, which should be determined in the second step of the DMD algorithm, is obtained by the convex optimization framework.

### 3.2. Dominant Modes Selection Strategy

Reconstruction matrix of DMD is obtained by a superposition of $\phi_{i}\exp(\varpi_{i}\Delta t)b_{i}$, and $\varpi_{i}$ is continuous-time eigenvalues of the similarity matrix $\tilde{A}$, which constitutes a diagonal matrix. Every single $\varpi_{i}$ corresponds to a DMD mode $\phi_{i}$. In this part, we introduce a method to select optimal dominant modes for DMD with two steps. Firstly, subtract modes relating to $\left\|\varpi_{i}\right\|=\left\|\ln(\lambda_{i})/\Delta t\right\|\approx 0$ from all the dynamic features, and then define a hierarchical application of basic DMD algorithm.

Step 1. By the construction of DMD methodology, $b=\Phi^{\Gamma }X$, which means that ϕb renders the columns of X with a dimensionality reduction. Thus, the diagonal matrix of frequencies $\lambda_{i}$ dictates how the first period of signal X gets altered over time to reconstruct the subsequent periods. It becomes apparent that any period of the signal that does not change or changes very slowly in time must have an associated Fourier mode located near the first period with the frequency $\left\|\varpi_{i}\right\|\approx 0$. We regard the slow oscillations as the background modes (noises) by setting a threshold ξ, and produce a representation of the low-frequency reconstruction matrix $X_{DMD}^{\left\|\varpi_{i}\right\|\leq \xi }$ and remaining frequency reconstruction matrix $X_{DMD}^{\left\|\varpi_{i}\right\|>\xi }$ of the form:

(17) $$X_{DMD}=X_{DMD}^{\left|\varpi_{i}\right|\leq \xi }+X_{DMD}^{\left|\varpi_{i}\right|>\xi }=\sum_{\left|\varpi_{i}\right|\leq \xi }\phi_{i}\exp(\varpi_{i}\Delta t)b_{i}+\sum_{\left|\varpi_{i}\right|>\xi }\phi_{i}\exp(\varpi_{i}\Delta t)b_{i}.$$

This observation makes it possible to pick out background modes from all the dynamic features with DMD, thus we only take the remaining frequency reconstruction matrix into account in this first step; thus,

(18) $$X_{DMD}\approx X_{DMD}^{\left|\varpi_{i}\right|>\xi }=\sum_{\left|\varpi_{i}\right|>\xi }\phi_{i}\exp(\varpi_{i}\Delta t)b_{i}.$$

Step 2. A hierarchical DMD is introduced to split the reconstructed signal with several levels, inspired by the idea of wavelet packet decomposition. Mathematically, we divide the solution of the DMD approximate solution of Equation (18) into slow modes and fast modes, by splitting the number of $\varpi_{i}>\xi$ at its median:

(19) $$\begin{array}{ll} {X_{DMD}}^{\left(1\right)} & =\sum_{i=1}^{r_{1}}{\phi_{i}}^{\left(1\right)}\exp({\varpi_{i}}^{\left(1\right)}\Delta t){b_{i}}^{\left(1\right)}\\ & =\sum_{i=1}^{r_{1}/2}{\phi_{i}}^{\left(1\right)}\exp({\varpi_{i}}^{\left(1,1\right)}\Delta t){b_{i}}^{\left(1\right)}+\sum_{i=(r/2+1)}^{r_{1}}{\phi_{i}}^{\left(1\right)}\exp({\varpi_{i}}^{\left(1,2\right)}\Delta t){b_{i}}^{\left(1\right)}\\ & ={X_{DMD}^{S}}^{\left(1\right)}+{X_{DMD}^{F}}^{\left(1\right)} \end{array},$$

where the symbol (1) at the top right corner of ${X_{DMD}}^{\left(1\right)}$ represents the first level, and $r_{1}$ equals the optimal rank r minus the number of $\left\|\varpi_{i}\right\|\leq \xi$. ${X_{DMD}^{S}}^{\left(1\right)}$ and ${X_{DMD}^{F}}^{\left(1\right)}$ are the slow modes matrix and fast modes matrix, respectively. Then, we apply the second segmentation on each of them:

(20) $$\begin{array}{lll} {X_{DMD}}^{\left(2\right)} & = & \sum_{i=1}^{r_{2}^{1}}{\phi_{i}}^{\left(2,1\right)}\exp({\varpi_{i}}^{\left(2,1\right)}\Delta t){b_{i}}^{\left(2,1\right)}+\sum_{i=r_{2}^{1}+1}^{r_{2}^{2}}{\phi_{i}}^{\left(2,2\right)}\exp({\varpi_{i}}^{\left(2,2\right)}\Delta t){b_{i}}^{\left(2,2\right)}+\\ & & \sum_{i=1}^{r_{2}^{3}}{\phi_{i}}^{\left(2,3\right)}\exp({\varpi_{i}}^{\left(2,3\right)}\Delta t){b_{i}}^{\left(2,3\right)}+\sum_{i=r_{2}^{3}+1}^{r_{2}^{4}}{\phi_{i}}^{\left(2,4\right)}\exp({\varpi_{i}}^{\left(2,4\right)}\Delta t){b_{i}}^{\left(2,4\right)}\\ & = & {X_{DMD}^{SS}}^{\left(2,1\right)}+{X_{DMD}^{FS}}^{\left(2,2\right)}+{X_{DMD}^{SF}}^{\left(2,3\right)}+{X_{DMD}^{FF}}^{\left(2,4\right)} \end{array},$$

where the symbol (SS) at the top right corner represents slow modes in the first level and also the same in the second level, while (SF) represent fast modes in the first level and slow modes in the second level.

The solution (20) can be made more precise by accounting for more numbers of segmentation; here, we say level (L). The arbitrary *L*-th DMD modes can be presented as follows:

(21) $${X_{DMD}}^{\left(L\right)}=\sum_{l}^{L}\sum_{j}^{J}\sum_{i=1}^{r_{l}}{\phi_{i}}^{\left(l,j\right)}\exp({\varpi_{i}}^{\left(l,j\right)}\Delta t){b_{i}}^{\left(l,j\right)},$$

where l=1,2,⋯,L represents the decomposition levels, $j=1,2,\cdots ,2^{l-1}$ represents the number of bins per level, and $r_{l}$ represents the truncation rank of l level.

**Figure 1:**
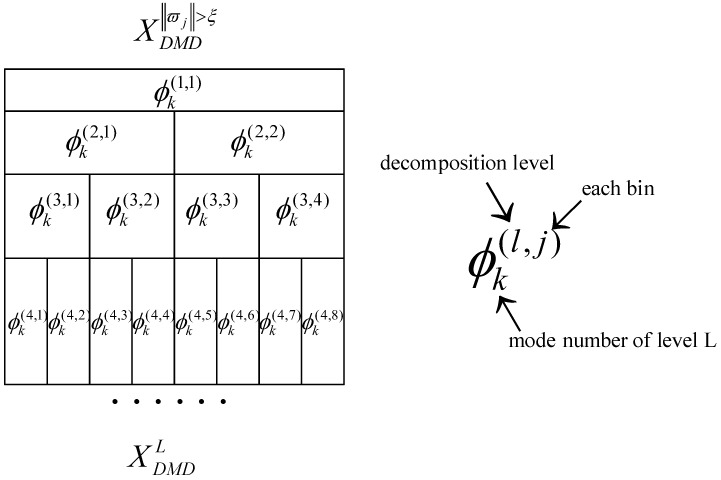
Illustration of the hierarchical DMD solution.

Figure 1 demonstrates the process in terms of the multiresolution DMD solution. Each ${X_{DMD}}^{\left(l,j\right)}$ can be obtained in each level l and each bin j by linear operator $A^{\left(l,j\right)}$:

(22) $$Y^{\left(l,j\right)}=A^{\left(l,j\right)}X^{\left(l,j\right)}.$$

The solution minimizes the error yielding the least-square fit. It can be given similarly by Formula (5).

As in the fifth step of DMD core algorithm in Section 2, Fourier transform can be applied on the recuperative signal for spectrogram with each ${X_{DMD}^{}}^{\left(l,j\right)}$, determining whether there is a failure frequency.

Rewrite the Equation (21) as follows:

(23) $$\begin{array}{ll} {X_{DMD}}^{\left(L\right)} & =\sum_{l}^{L}\sum_{j}^{J}\sum_{i=1}^{r_{l}}g^{\left(l,j\right)}{\phi_{i}}^{\left(l,j\right)}\exp({\varpi_{i}}^{\left(l,j\right)}\Delta t){b_{i}}^{\left(l,j\right)}\\ & =g^{\left(l,j\right)}\Phi^{\left(l,j\right)}\exp(\Omega^{\left(l,j\right)}t)b^{\left(l,j\right)} \end{array}.$$

Define that $g^{\left(l,j\right)}=1$ when there is a failure frequency for ${X_{DMD}^{}}^{\left(l,j\right)}$, accordingly, $g^{\left(l,j\right)}=0$ when there is no failure frequency. $g^{\left(l,j\right)}$ acts as a sifting operation for each bin j at level l.

**Figure 2:**
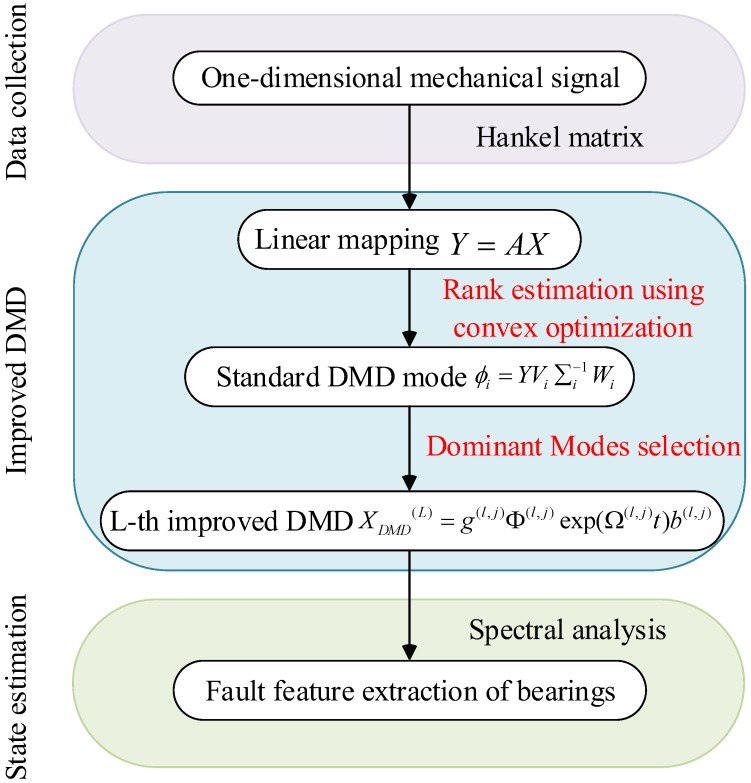
A flow chart of applying improved DMD.

Ultimately, the flowchart of the improved DMD method is shown in Figure 2.

## 4. Bearing Fault Signal Processing with Improved DMD

### 4.1. Analysis of Simulated Signal

There are many kinds of dynamic models simulating bearing fault signal. Comparatively speaking, the model first established by McFadden [26] and later refined by Randall [27] can better reflect the characteristics of rolling bearing vibration. According to the structure characteristics, short-term impact signal should be produced when bearing parts revolve through the fault location. The impact motivates the bearing system oscillating by its natural frequency with high frequency attenuation. Define *t* as a period of two intervals, S(t) as the natural frequency oscillation function, and $A_{k}$ as the *i*-th amplitude of shock response. The interference of additive noise $n_{t}$ (zero mean additive background noise) should be taken into consideration along with the simulation signal in view of the poor working environment of rolling bearings. Therefore, the mathematical model of fault rolling bearing can be described as:

(24) $$\{\begin{array}{l} f(t)=\sum_{k=1}^{M}A_{k}S(t-kT-\tau_{k})+n(t)\\ A_{k}=a_{k}\cos(2\pi f_{m}t+\varphi_{A})+c_{A}\\ S(t-kT-\tau_{k})=\exp[-B(t-kT-\tau_{k})\cdot \sin[2\pi f_{n}(t-kT-\tau_{k})+\gamma ] \end{array},$$

where $a_{k}$ is the *k*-th impact energy; γ and $\varphi_{A}$ are the initial phases; $f_{m}$ is the modulation frequency; B is the attenuation coefficient depended on the bearing system; $\tau_{k}$ is the time lag from its mean period due to the presence of tiny fluctuation, and $c_{A}$ is a random constant.

**Figure 3:**
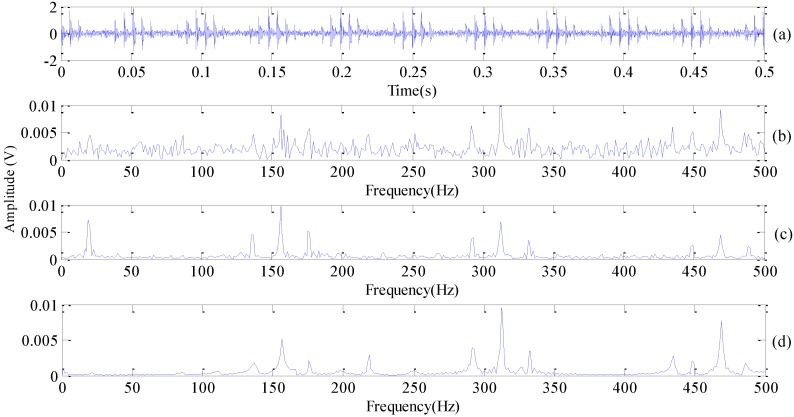
Improved DMD analysis with simulation signal of bearing inner race (**a**) time domain fault simulation signal; (**b**) FFT spectrum; (**c**) envelope spectrum; (**d**) frequency spectrum with improved DMD.

Most frequently, the fault of rolling bearing appears as the damage in outer race, inner race, and/or the surface of rolling elements. Generally, three common types of faults on outer race, inner race and rolling elements can be modeled as $f_{m}=0,$ $f_{m}=f_{r}$, $f_{m}=f_{c}$, respectively. Here, $f_{r}$ and $f_{c}$ are denoted as rotational frequency and cage frequency. The time-domain fault simulation signal of the inner race is plotted in Figure 3a, added by the Gaussian white noise so the SNR ratio is 6. The parameters selection of Equation (24) is listed in Table 1.

We choose ξ=0.04 (retain 90% of $\varpi_{i}$) and take the decomposing level L as 2. Frequency spectrum with improved DMD plotted in Figure 3d, while FFT frequency and envelope spectrum are also plotted for comparison in Figure 3b,c, respectively. Inner race fault frequency can be identified, but the modulation frequency (FM) is not clear connected to the intuition with the FFT frequency spectrum. Inner race frequency and rotational modulation frequency are visibly presented in the envelope spectrum, but there is still a certain amount of noise frequency surrounding the principal frequencies. By adopting our improved DMD, not only can the frequency of inner race fault be clearly visualized, but there is also a little amount of noise outlined in Figure 3d compared with Figure 3c.

**Figure 4:**
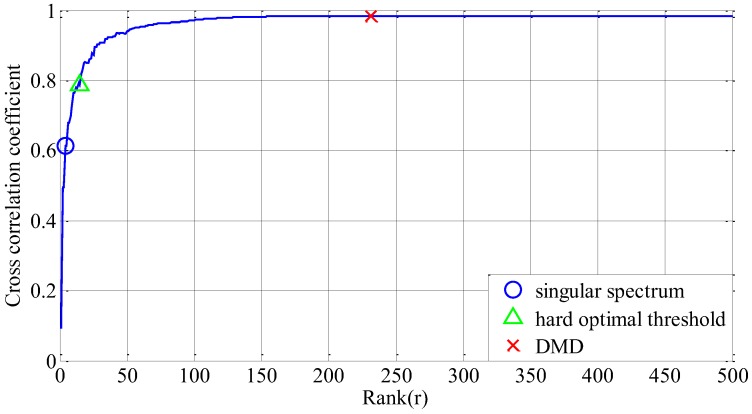
Cross correlation coefficient between DMD reconstruction signal and original simulation signal with different truncation rank *r*. Cross correlation coefficient is first ascending and then slightly declining. The rank (r=4) in blue circle point obtained by singular spectrum; the rank (r=13) with the green triangle point obtained by the hard optimal threshold; the rank (r=231) with a red cross mark obtained by our method.

Variations of cross correlation coefficient along with the truncated rank order are shown in Figure 4. The best truncation rank number of SVD, hard optimal threshold and convex optimization DMD were also marked on the curve. It can be concluded that the sparse optimization framework outperforms the other methods in the quality of signal recovery.

**Figure 5:**
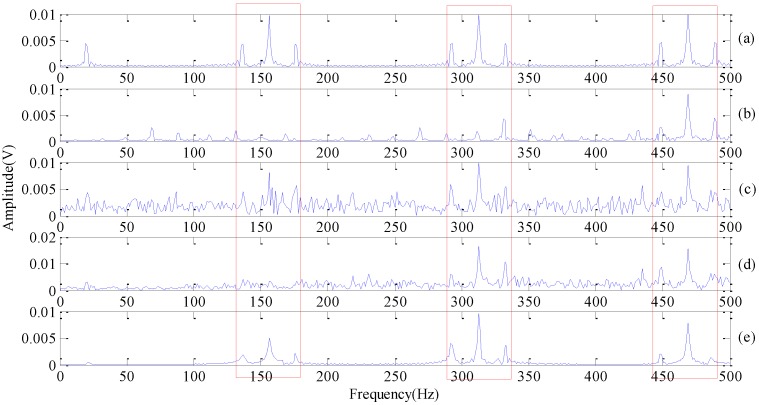
Frequency spectrum with different methods (**a**) FFT of noise-free signal; (**b**) SVD; (**c**) WT; (**d**) EMD; (**e**) improved DMD.

Comparisons between improved DMD with other conventional spectral analysis methods are characterized in Figure 5. Figure 5a is the frequency spectrum of the original situation signal without noise taking by FFT. Figure 5b is the result of SVD, and we select the first four singular values for signal reconstruction. Figure 5c is the multi-scale WT signal spectrum; here, we use wavelet function db4 with three scale wavelet decomposition [5], and choose the high frequency component of the second layer for signal reconstruction. Figure 5d is the frequency spectrum by applying FFT on superposed IMFs of EMD, and we choose IMFs of 1, 4, 5, 6 for superposition. Figure 5e was obtained by our improved DMD method, while ξ=0.04, L=3. SNR of the reconstruction signal is calculated as 7.8, 9.5, 6.6, 14.2 from Figure 5b–e successively. Our method has a tremendous advantage in both denoising and fault feature extraction.

### 4.2. Analysis of Measured Signal

**Figure 6:**
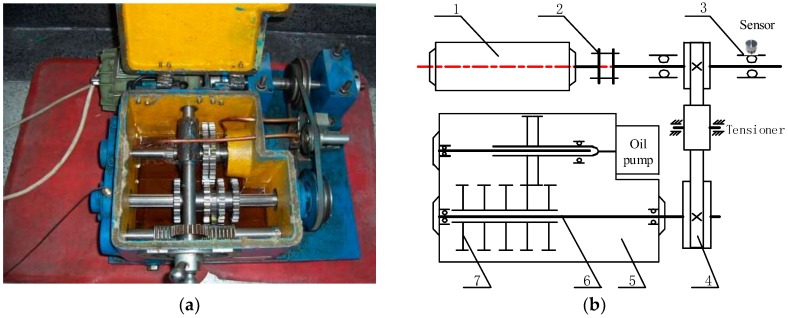
Gear-bearing comprehensive failure device: (**a**) test rig general view; (**b**) structure diagram of test device: 1—motor, 2—coupling, 3—test rolling bearing from 6207 series, 4—belt pulley, 5—housing, 6—transmission shaft, 7—gear.

Vibration signals of the bearing system in an industrial spot are more complex than the dynamic simulation signal [28]. To verify the effect of fault features extraction, the improved DMD algorithm is applied on real environment fault signals. An integrated fault apparatus of gear-bearing transmission is shown in Figure 6. The high-speed shaft is driven by an AC motor (550 w, 220 v/50 Hz) with a belt, and the speed of the motor is 1450 r/min. A corrosion pit with the depth of 1.0 mm was fabricated by electric spark processing in the out race of the testing bearing (deep groove ball bearing, bearing number 6207). An acceleration sensor (PCB–352C33, Depew, NY, USA) is installed on the bearing pedestal along the vertical direction. The vibration signal is then obtained by a machinery health analyzer CSI2130 (Emerson, St. Louis, MO, USA) with sampling frequency of 16,384 Hz. The number of rolling elements is Z=9, and ball diameter is d=11.1 mm, pitch diameter of the testing bearing is D=53.5 mm. Thus, rotation frequency and outer ring fault frequency can be calculated as 24.17 Hz and 87.01 Hz, respectively [29].

**Figure 7:**
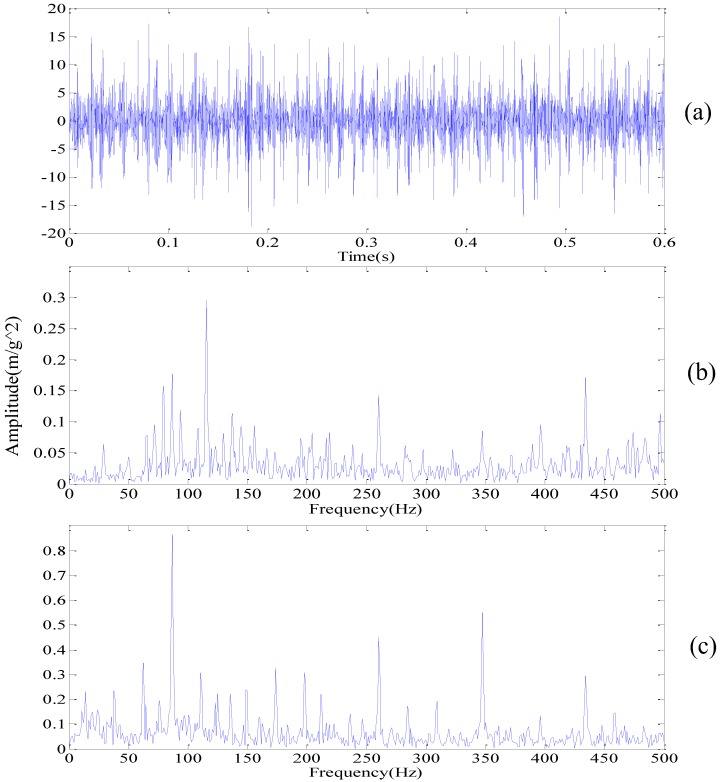
The measured signal in the time domain and frequency spectrum: (**a**) time domain; (**b**) FFT spectrum; (**c**) envelope spectrum.

A total number of 10,000 samples, shown in Figure 7a in the time domain, are analyzed with improved DMD. The FFT spectrum is shown in Figure 7b. Pang [30] proved the superiority of the envelope spectrum over the FFT in the incipient rolling bearing fault diagnosis. For comparison, the envelope spectrum of the measured signal is shown in Figure 7c.

**Figure 8:**
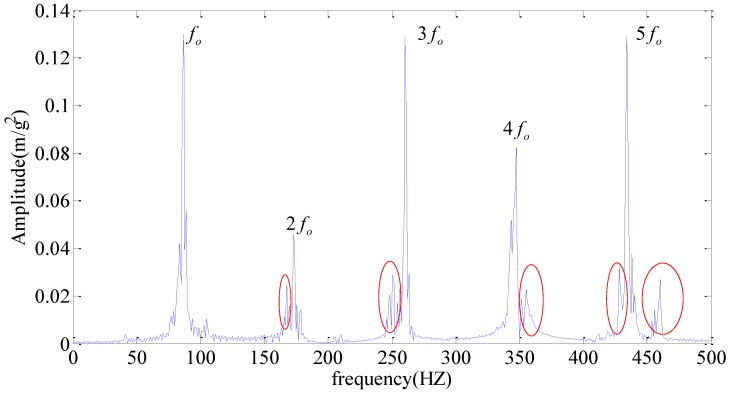
Frequency spectrum of WPT.

Obviously, we can’t judge whether there is a bearing fault from Figure 7b for a large amount of interference frequencies presented in the frequency spectrum. Figure 7c is slightly better than Figure 7b, but there are still some noise frequencies presented in the frequency spectrum. Meanwhile, in order to illustrate the effectiveness of our method, the frequency spectrum of WPT is applied and the results are shown in Figure 8. Here, we use wavelet function db15 with eleven layers of decomposition. Though it is obvious that the fault frequency $f_{o}$ and its harmonics ($2f_{o}$, $3f_{o}$, $4f_{o}$, $5f_{o}$) can be identified in Figure 8, where $f_{o}$ is the frequency of the outer race, the noise is still present in the acquired signal, as we can see in the red elliptical mark.

**Figure 9:**
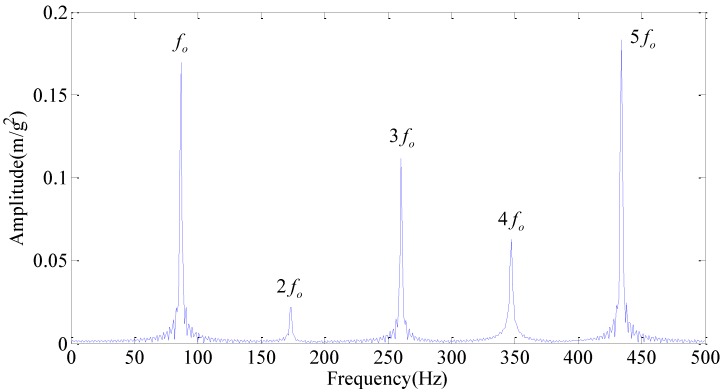
The result provided by improved DMD.

Subsequently, we apply our improved DMD method on the measured signal with chosen parameters m=5000,ξ=0.05,L=7. The non-fault signal modes are filtered out and the useful signals with characteristic frequencies are retained by examining each bin (total sixty-four pins) of the seventh level. Then, the reconstruction matrix can be obtained by Formula (23). Figure 9 shows the spectrum of our improved DMD method. Obviously, not only are the fault frequency $f_{o}$ and its harmonics ($2f_{o}$, $3f_{o}$, $4f_{o}$, $5f_{o}$) easy to be identified, but there is also little interference frequency close to them. That is to say, we are more confident to determine that the outer race of the testing rolling bearing has failed, which is highly consistent with the actual situation.

## 5. Conclusions

This paper puts forward an improved DMD decomposition algorithm, which successfully applied on the bearing fault signal. Main solution of our work includes the following two aspects: (1) optimal rank truncation number of the similarity matrix is obtained by using convex optimization through introducing a parameterized non-convex penalty function. Our rank determinative method is more perfectible than others in feature extraction with standard DMD algorithms; (2) in order to archive the dominant modes selection strategy, we firstly subtract slow oscillations modes by setting a threshold, and then define a hierarchical application of the basic DMD algorithm. We apply our improved DMD framework both on simulated and measured signals for roller bearing signal fault diagnosis. SNR is better than other classical noise reduction methods after the bearing simulation signal is processed by our improved DMD algorithm. In addition, the frequency spectrum of the reconstruction signal is more readable and interference-free. Results show that our method has a good application prospect in denoising and feature extraction with a mechanical signal.

## Figures and Tables

**Table 1:** The parameters selection with simulation signal of bearing inner race.

$a_{k}$	γ	$\varphi_{A}$	$f_{r}(Hz)$	B	$\tau_{k}$	$c_{A}$	$Inner\;Race\;f_{i}(Hz)$
3	0	0	20	800	0.01	1	156
